# Building pyramids against the evolutionary emergence of pathogens

**DOI:** 10.1098/rspb.2023.1529

**Published:** 2024-03-13

**Authors:** Sylvain Gandon, Martin Guillemet, François Gatchitch, Antoine Nicot, Ariane C. Renaud, Denise M. Tremblay, Sylvain Moineau

**Affiliations:** ^1^ CEFE, Univ Montpellier, CNRS, EPHE, IRD, Montpellier, France; ^2^ Département de biochimie, de microbiologie et de bio-informatique, Faculté des sciences et de génie, Université Laval, Quebec city, Canada G1V0A6; ^3^ Félix d'Hérelle Reference Center for Bacterial Viruses, Université Laval, Québec City, Canada G1V 0A6

**Keywords:** evolutionary emergence, epidemiology, demographic stochasticity, CRISPR resistance, bacteriophage, infectious disease control

## Abstract

Mutations allowing pathogens to escape host immunity promote the spread of infectious diseases in heterogeneous host populations and can lead to major epidemics. Understanding the conditions that slow down this evolution is key for the development of durable control strategies against pathogens. Here, we use theory and experiments to compare the efficacy of three strategies for the deployment of resistance: (i) a *mixing* strategy where the host population contains two single-resistant genotypes, (ii) a *pyramiding* strategy where the host carries a double-resistant genotype, (iii) a *combining* strategy where the host population is a mix of a single-resistant genotype and a double-resistant genotype. First, we use evolutionary epidemiology theory to clarify the interplay between demographic stochasticity and evolutionary dynamics to show that the *pyramiding* strategy always yields lower probability of evolutionary emergence. Second, we test experimentally these predictions with the introduction of bacteriophages into bacterial populations where we manipulated the diversity and the depth of immunity using a Clustered Regularly Interspaced Short Palindromic Repeats-CRISPR associated (CRISPR-Cas) system. These biological assays confirm that pyramiding multiple defences into the same host genotype and avoiding combination with single-defence genotypes is a robust way to reduce pathogen evolutionary emergence. The experimental validation of these theoretical recommendations has practical implications in various areas, including for the optimal deployment of resistance varieties in agriculture and for the design of durable vaccination strategies.

## 1. Introduction

The spread of pathogen epidemics is driven by the composition of host populations and, in particular, by the fraction $f_{R}$ of resistant hosts. Larger values of $f_{R}$ generate ‘herd immunity’ in well-mixed populations because a randomly chosen susceptible host is expected to be surrounded by many resistant neighbours. Since resistant hosts cannot be successfully infected, their presence shields susceptible individuals from the risk of being infected and reduce the spread of a given pathogen. In fact, the epidemic is expected to stop growing when $f_{R}>1-1/R_{0}$, where $R_{0}$ refers to the basic reproduction ratio of the pathogen. This theoretical framework provides key guidelines for the deployment of control measures like vaccination [1,2] or the deployment of resistant varieties of crops in agriculture [3,4].

The efficacy of these control strategies, however, is challenged by the potential acquisition of escape mutations allowing the pathogen to infect a resistant host. Whether those variants will appear, establish and spread depends on multiple evolutionary forces, including the composition of the host population. For instance, a larger fraction $f_{R}$ of resistant hosts is expected to reduce the growth rate of the wild-type pathogen and, consequently, to limit the influx of escape mutations. But a larger fraction $f_{R}$ of resistant hosts is also expected to increase the fitness benefit associated with an escape mutation. This will increase the probability of establishment of a given mutation (i.e. lower risk of stochastic extinction when rare) and it will also increase the speed at which this variant will spread. The balance between these two opposite effects may thus result in a higher risk of pathogen adaptation for intermediate frequency of resistance. Hence, a better understanding of the influence of the host population composition on pathogen adaptation may help identify more durable control strategies.

Many theoretical studies have explored complex ecological scenarios to evaluate the impact of various strategies for the deployment of host-resistant genotypes across space and time in agriculture [3,5–14]. In particular, several studies contrasted the efficacy of *mixing* multiple single host-resistant genotypes with the efficacy of *pyramiding* multiple resistant genes within a single genotype. Earlier models did not incorporate demographic feedbacks or any influence of demographic stochasticity and focused on the long-term deterministic evolutionary outcomes. Under these conditions the *mixing* strategy can outperform the *pyramiding* strategy because the former strategy can prevent the spread of pathogens carrying multiple escape mutations [5,6]. More recent studies challenged this guidance and relied on simulation models that highlight the importance of epidemiology, demographic stochasticity, and spatial structure on both the epidemiology and the evolution of the pathogen [9–11,14,15]. Taken together, the available theoretical literature may appear confusing because distinct studies make different recommendations on the optimal strategy for the deployment of resistance against a pathogen [16]. This confusion stems from the different assumptions of the models (e.g. with or without demography, with or without stochasticity) but also on the different optimality criteria used to identify the most effective pathogen control strategies (e.g. no evolution of multi-escape mutations, minimal disease incidence) [8,17,18]. Besides, experimental studies needed to evaluate the durability of control strategies against pathogens are notoriously difficult to carry out, particularly in agriculture [8,13,19]. This lack of experimental validation does not help to elucidate the pros and cons of distinct deployment of resistance strategies.

Here, we develop a joint theoretical and experimental approach to analyse the durability of different strategies for the deployment of host resistance. We focus on a very specific quantity to evaluate the efficacy of a control strategy: the probability of pathogen emergence with (or without) adaptation. This quantity provides a relevant measure of control efficacy because it accounts for both short-term (epidemiological timescale) and long-term (evolutionary timescale) dynamical processes [20]. Experimental measurements of evolutionary emergence, however, are challenging because the stochastic nature of pathogen extinction requires a large number of replicate populations to measure the probability of emergence. These experiments also require the ability to manipulate the composition of the host population and to track the evolution of the pathogen population. These hurdles can be overcome by studying the evolutionary emergence of virulent bacteriophages in bacterial populations that use the adaptive CRISPR (Clustered Regularly Interspaced Short Palindromic Repeats) immunity to prevent phage infections.

CRISPR-Cas adaptive immune systems are widespread among many bacteria and archaea. A CRISPR immunity phenotype is genetically encoded by a so-called CRISPR locus (Clustered Regularly Interspaced Short Palindromic Repeats)—an array of short repetitive and unique nucleotide sequences (‘repeats’ and ‘spacers’, respectively). ‘Spacers’ are derived from (foreign) genetic elements, such as viral genomes, and provide immunity to re-infection based on recognition (via a short CRISPR RNA) and cleavage (by a Cas nuclease) of the cognate sequence (known as ‘protospacer’). Crucially, a virus can adapt to CRISPR-Cas immunity if it acquires escape mutations in the protospacer targeted by the host [21–23].

This natural microbial system offers a unique opportunity to study the dynamics of viral adaptation in heterogeneous host populations: (i) many replicates can be carried out simultaneously using bacteria and phages in 96-well plates [20], (ii) CRISPR immunity provides a very convenient way to manipulate both the *diversity* of host immunity (different bacteria derived from the same population can carry different ‘spacers’ in their CRISPR array [20,24]) and the *depth* of host immunity (multiple ‘spacers’ can be stacked within the CRISPR array of the same multiresistant bacterium [25]), (iii) the most common mechanism of phage adaptation to CRISPR-based immunity is well documented: virulent phages escape CRISPR resistance through mutation in their target sequence (the ‘protospacer’) [21–23,26].

In the next sections we present the theoretical framework used to compute the probability of evolutionary emergence of pathogens after being introduced in a heterogeneous host population. We use this model to understand the effect of multiple factors on the fate of the pathogen: (i) the number of viruses introduced, (ii) the proportion of resistant hosts, (iii) the diversity and the depth of immunity of resistant hosts. This allows us to contrast the influence of different strategies of resistance deployment on the probability of pathogen evolutionary emergence. In a second step, we manipulated the heterogeneity of bacterial CRISPR immunity to experimentally test the validity of our theoretical predictions on the evolutionary emergence of phage populations.

## 2. Material and methods

### (a) Theory

**Figure 1.**
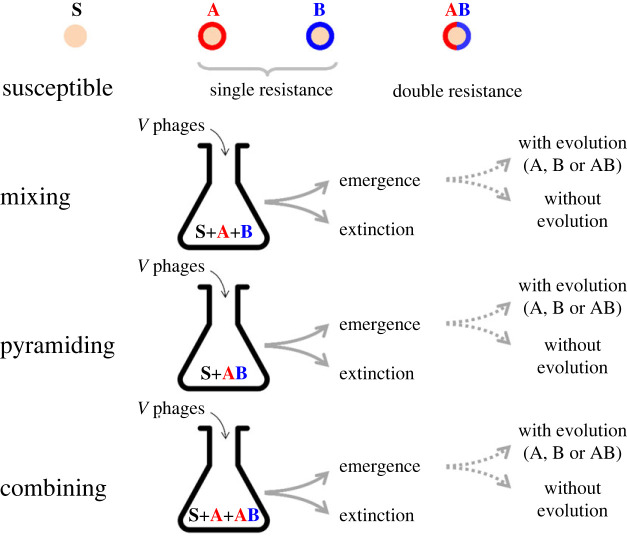
Schematic representation of the *mixing*, *pyramiding* and *combining* scenarios. In each scenario the host population is a mix of a proportion of susceptible bacteria (S) and a proportion $f_{R}$ of resistant bacteria (A, B and AB). In our experiment we used $f_{R}=0.9$. The composition of the population of resistant bacteria differs between the *mixing* (1 : 1 mix of two single-resistant hosts A + B), the *combining* (1:1 mix of a single-resistant host, A (combining A) or B (combining B), and a double-resistant host AB), and the *pyramiding* (a double-resistant host AB) scenarios. After the inoculation of *V* phages the viral population may either go extinct or produce an epidemic. The virus epidemic may either result from the replication of the ancestral virus (no evolution) or in the additional replication of phage genotypes carrying escape mutations against A, B or AB. We carried out these experiments in 96-well plates that allowed us to replicate our inoculation experiment in 96 host populations (each replicate population was 200 µl). After an overnight incubation of the cultures (22 h) we measured (i) the occurrence of phage epidemics (i.e. emergence) by plating a fraction (2 µl) of each replicate population on a lawn of sensitive cells (*Streptococcus thermophilus* DGCC 7710) and (ii) the presence of escape phage mutants (i.e. evolutionary emergence) by plating a fraction (2 µl) of each replicate population on a lawn of singly resistant (A or B) or doubly resistant (AB) bacteria (see electronic supplementary material).

Pathogen emergence is defined as the ability to escape early extinction and thus to initiate an epidemic after the introduction of a small quantity of pathogens in the host population. This probability of emergence depends both on the host (e.g. proportion and diversity of resistant hosts) and the pathogen (e.g. inoculum size, viral mutation rate, genetic composition) [20,27–29]. For a pathogen to emerge, we assume that the host population contains a fraction of individuals fully susceptible to the pathogen while the remaining fraction $f_{R}$ of the population is resistant. Resistance is assumed to be perfect (a virus cannot infect a resistant host without a matching escape mutation) but we previously showed in [20] how it is possible to expand this analysis to imperfect immunity. Among the resistant hosts, we consider three alternative scenarios (figure 1):

(i) a *mixing* scenario, in which the resistant fraction of the population is a mix of two single-resistance genotypes (A + B) aiming at distinct pathogen *target sites* (a target site is defined here as a region of the pathogen genome recognized by immune effectors and where a mutation or a deletion may allow escape recognition by host immunity). We allow the frequency of the two resistant hosts to vary and $f_{A}$ refers to the frequency of the resistant host A among the resistant hosts ($f_{B}=1-f_{A}$ is the frequency of the resistant host B among the resistant hosts),
(ii) a *pyramiding* scenario, in which the resistant fraction of the population is monomorphic with a double-resistance genotype (AB),
(iii) a *combining* scenario, in which the resistant fraction of the population results from a mix of single-resistance genotypes (say A) and a double-resistance genotype (AB). We allow the frequency of the two resistant hosts to vary and $f_{A}$ refers to the frequency of resistant host A among the resistant hosts ($f_{\mathrm{AB}}=1-f_{A}$ is the frequency of the resistant host AB among the resistant hosts). Note that the *pyramiding* scenario is a limit case of the *combining* scenario when $f_{A}=0$.

Even if host resistance is assumed to be perfect the pathogen can evade recognition by acquiring escape mutations in the corresponding immunity target sites. Therefore, a pathogen with escape mutation *i* can infect a fraction $(1-f_{R})+f_{R}P_{i}$ of the total host population, where $P_{i}=\sum_{h\in \{A,B,AB\}}\;f_{h}P_{i}^{h}$ is the fraction of the resistant hosts that can be infected by the pathogen with genotype *i* and $P_{i}^{h}$ measures the ability of the pathogen genotype *i* to infect the host genotype h∈{∅,A,B,AB}. In the *pyramiding* scenario, pathogens with less than two escape mutations can only infect a fraction $\left(1-f_{R}\right)$ of the total host population and only pathogens with two escape mutations can infect all the hosts. In the *mixing* and in the *combining* scenarios, the fitness of a single escape mutant depends on the identity of the single-resistance genotype in the host population and the composition of the resistant population (see electronic supplementary material).

We further assume a classic birth-death process to model the epidemiological dynamics where a host infected with a pathogen that does not carry escape mutations spreads this pathogen in a fully susceptible host population at rate *b* and dies at rate *d*. Host resistance prevents infection and may thus affect the effective birth rate, but without affecting *d*. Whereas escape mutations may allow the pathogen to infect a larger fraction of the host population, they also carry a fitness cost *c* which causes pathogens with *i* escape mutations to reproduce at rate $b_{i}=b{\left(1-c\right)}^{i}$. The probability of acquiring an escape genotype i∈{A,B,AB} by mutation is noted $\mu_{i}$ and may vary among target sites. Note that different genetic changes in the protospacer could allow the virus to escape recognition by CRISPR-Cas immunity [30]. This increases the mutation rate towards an escape phenotype, but we do not need to distinguish these different mutations to model the dynamics of the virus. Crucially, the acquisition of two escape mutations requires two independent changes of the phage genome, which is expected to occur at a much smaller rate than the rate of single escape mutations: $\mu_{AB}\approx \mu_{A}\mu_{B}\ll \mu_{A},\mu_{B}$. For the sake of simplicity, we assume that escape mutations are fixed and cannot revert to the ancestral types. These reversions to the wild-type target are expected to have a negligible effect on the probability of evolutionary emergence when the target site mutation rate remains small [20,31].

We detailed in the electronic supplementary material, how we compute the probability of emergence (with or without viral evolution) after the introduction of an inoculum of *V* phage particles in a heterogeneous bacterial host population. We also derive approximations for the probability of evolutionary emergence inspired from models of evolutionary rescue. Those approximations help to contrast the effects of the composition of the host population on the risk of evolutionary emergence.

### (b) Experiments

We used the Gram-positive bacterial strain *Streptococcus thermophilus* DGCC 7710 which is susceptible to the virulent phage 2972. We also used three CRISPR-resistant clones (also referred to as bacteriophage-insensitive mutants: BIMs) that were derived from *S. thermophilus* DGCC 7710 and differ only in their CRISPR arrays (electronic supplementary material, tables S1 and S2). Two of these clones carried a single additional spacer (strains A and B) targeting the genome of phage 2972, while the remaining clone carried a combination of these two spacers (strain AB) precisely obtained using the approach developed by Hynes *et al*. [25]. The addition of a single spacer in the CRISPR1 array of *S. thermophilus* DGCC 7710 provides a robust resistance against infection by the wild-type virulent phage 2972 [21–23] (electronic supplementary material, table S2). The rate at which phage 2972 acquires a mutation allowing to escape CRISPR immunity was found to be approximately equal to 2.8 × 10^−7^ mutations/locus/replication [32]. The acquisition of a single escape mutation may or may not yield significant fitness costs for the phage [22,32].

We monitored the dynamics of the phage population after introducing an inoculum of *V* viruses in each well of a 96-well plate containing 200 µl of replicate bacterial populations with a proportion $f_{R}=90\text{\%}$ of resistant cells and $1-f_{R}=10\text{\%}$ of susceptible cells. This virus inoculum was sampled from a lysate obtained after amplifying a single plaque of the wild-type phage 2972 on *S. thermophilus* DGCC 7710 (the initial frequency of single and double escape mutants was estimated in electronic supplementary material, table S3: 3.7 × 10^−6^ and 1.7 × 10^−6^ against single resistance A and B, respectively, but we did not detect double escape mutants). We manipulated the composition of the resistant bacterial population to produce three experimental treatments to test the predictions of the theoretical model (figure 1): (i) *mixing* (strains A and B in equal frequency), (ii) *pyramiding* (only strain AB), (iii) *combining* strains A and AB in equal frequency (combining A) or *combining* strains B and AB in equal frequency (combining B). After an overnight incubation (22 h) we quantified the abundance and the evolution of the phages after spotting a fraction of each replicate (2 µl) on a lawn of the different bacterial strains to measure: (i) the presence/absence of phages using a lawn of susceptible cells, (ii) the presence/absence of escape mutations in the phage population using lawns of single-resistance bacteria (A or B) and a lawn of double-resistance bacteria (AB) [20,32].

We used logistic regression models with the presence/absence on susceptible bacteria (or on resistant bacteria) as the response variable as a function of the inoculum size and the composition of the host population (see electronic supplementary material).

## 3. Results

### (a) Emergence and evolutionary emergence

**Figure 2.**
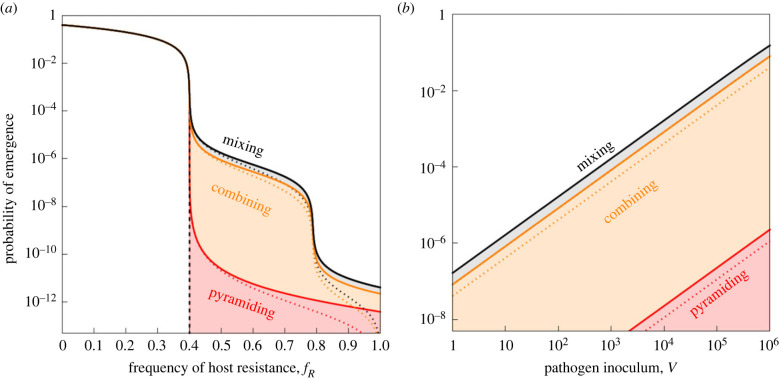
Theoretical predictions for pathogen emergence under the *mixing* (black), *combining* (orange) and *pyramiding* (red) scenarios. In (*a*) we show the effect of $f_{R}$, the fraction of resistant hosts in the host population, on the probability of pathogen emergence after the introduction of a single virus (V=1) with or without pre-existing mutations (full or dotted lines, respectively). The probability of emergence in the absence of pathogen evolution is indicated with the dashed black line. The colour shading refers to *evolutionary emergence* in the different scenarios (i.e. pathogen emergence resulting from pathogen evolution). In (*b*) we show the effect of *V*, the size of the virus inoculum, on the probability of pathogen emergence with or without pre-existing mutations when $f_{R}=0.7$. Other parameter values: b=1.66, d=1, c=0.01, $\mu =10^{-6}$, $p_{\emptyset }=1-p_{A}-p_{B}-p_{AB}$, $p_{A}=p_{B}=10^{-6}$, $p_{AB}=10^{-12}$ (without pre-existing mutations: $p_{\emptyset }=1$, $p_{A}=p_{B}=p_{AB}=0$).

We derive the probability of pathogen emergence after the introduction of an inoculum of *V* pathogens. This inoculum is sampled from a population where some phage genotypes may already carry escape mutations: $p_{i}$ refers to the frequency of genotype i∈{∅,A,B,AB}. In the following we focus mainly on scenarios where the frequencies of pre-existing escape mutations remain low (i.e. $p_{\emptyset }\approx 1$). Figure 2 shows the effect of the inoculum size and the frequency of resistance on pathogen emergence under different deployment strategies.

In the absence of pathogen mutations ($p_{\emptyset }=1$ and μ=0), the probability of pathogen emergence is equal to $P_{E}=1-{\left(\;f_{R}+1/R_{0}\right)}^{V}$ when $f_{R}+1/R_{0}>1$, where $R_{0}=b/d$ is the basic reproduction ratio of the pathogen [20]. As indicated with a dashed line in figure 2, this probability of pathogen emergence drops rapidly with the increase in the proportion of resistant hosts and pathogen emergence becomes impossible when $f_{R}>1-1/R_{0}$. Note that this threshold is independent of the deployment strategy because they all share the same value of $f_{R}$.

However, the pathogen population may avoid extinction through the acquisition of escape mutations. The term *evolutionary emergence* refers to these situations where emergence is consecutive to pathogen evolution [28,29]. In figure 2 we compare the probabilities of evolutionary emergence in a symmetric scenario where $f_{A}=1/2$ for increasing values of $f_{R}$ (figure 2*a*) and *V* (figure 2*b*). Crucially, the probability that the pathogen adapts to host resistance depends on the deployment of host resistance strategies and the *pyramiding* treatment always yields lower probability $f_{R}$$P_{EE}$ of evolutionary emergence. Indeed, in both the *mixing* and the *combining* treatments, the presence of a single-resistance genotype provides a ‘stepping stone’, allowing the virus to recover the ability to grow in the host population after the acquisition of a single escape mutation. Besides, the acquisition of this first escape mutation may allow the pathogen to acquire later on the ability to escape both types of resistance. The lower probability to acquire both escape mutations at the same time explains the step-like shape of the probability of emergence in figure 2 (see also electronic supplementary material, figure S1). As expected, pre-existing mutations always increase the probability of pathogen emergence and allow the pathogen population to escape extinction even in the extreme case where $f_{R}=1$ and no fully susceptible hosts are present in the host population (figure 2*a*).

**Figure 3.**
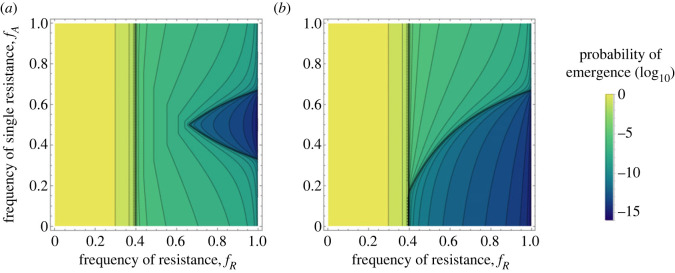
Theoretical predictions for pathogen emergence under (*a*) the *mixing* and (*b*) the *combining* scenarios. We show the effect of $f_{R}$, the fraction of resistant hosts in the host population, and $f_{A}$, the fraction of a single-resistant host in the resistant host population on the probability of pathogen emergence after the introduction of a single virus (V=1) and without pre-existing mutations ($p_{\emptyset }=1$, $p_{A}=p_{B}=p_{\mathrm{AB}}=0$). Other parameter values: b=1.66, d=1, c=0.1, $\mu =10^{-6}$.

We can generalize these results for asymmetric scenarios where $f_{A}\neq 1/2$. Interestingly, variations of $f_{A}$ have different effects in the mixing and combining treatments (figure 3). In the *mixing* treatment, the probability of emergence is minimized when $f_{A}$ is close to 1/2 and thus when the amount of diversity is maximized in line with the effect of diversity discussed in Chabas *et al.* [20]. In the *combining* treatment, the risk of emergence is minimized when because this is the case where all the resistant hosts carry two resistances (i.e. *pyramiding* treatment).

The influence of host composition on the probability of evolutionary emergence can be captured within the framework of evolutionary rescue models. This framework is relevant as soon as $f_{R}>1-1/R_{0}$ because the wild-type virus is doomed to go extinct when the proportion $f_{R}$ of resistant hosts leads to a negative growth rate of the wild-type virus population. We derive approximations for the probability of evolutionary emergence under the assumption that the viral mutation rate is small (electronic supplementary material). In the symmetric scenario (i.e. $f_{A}=1/2$) this yields:

3.1 $$\begin{array}{rl} \mathrm{Mixing}:P_{\mathrm{EE}}^{M}\approx & 2V\mu \left(1-\frac{d}{b(1-c)(1-f_{R}/2)}\right)+O(\mu^{2})\\ \mathrm{Combining}:P_{\mathrm{EE}}^{C}\approx & V\mu \left(1-\frac{d}{b(1-c)(1-f_{R}/2)}\right)+O(\mu^{2})\\ \mathrm{Pyramiding}:P_{\mathrm{EE}}^{P}\approx & O(\mu^{2}). \end{array}\}$$

This approximation captures both the effect of a larger inoculum size and the effect of treatment on $P_{\mathrm{EE}}$ illustrated in figure 2. Note that larger inoculum sizes are also expected to increase $P_{\mathrm{EE}}$ via the introduction of pre-existing mutants, not modelled in (3.1). The above approximation is particularly useful to discuss the effect of the composition of the host population. In particular, in the *mixing* strategy the $P_{\mathrm{EE}}$ is expected to be twice as large than in the *combining* strategy in the symmetric scenario. And both these strategies are expected to have higher $P_{\mathrm{EE}}$ than the *pyramiding* strategy because $\mu_{\mathrm{AB}}$ is assumed to be much smaller than $\mu_{A}$ and $\mu_{B}$.

### (b) Experiments

Increasing the size *V* of the virus inoculum increased the ability to observe the presence of phages on fully susceptible bacterial populations (Type II Anova: LR chi-square = 3744.2, d.f. = 1, *p* < 2.2 × 10^−16^) and reached its maximal value when $V>10^{3}$ (electronic supplementary material, figure S2). We found an effect of host treatment on the probability to detect phages on fully susceptible bacteria which is difficult to interpret because it interacts with the inoculum size (electronic supplementary material). Importantly, note that this treatment effect is not due to pathogen evolution since pathogen evolution is not detectable when $V<10^{3}$.

It is tempting to equate our measure of the presence of phages on susceptible bacteria with the probability of emergence $P_{E}$. Yet, as soon as the wild-type phages start to replicate, the proportion of susceptible bacteria is expected to drop and $f_{R}$ is expected to be ≈1 after the overnight culture. So, the presence/absence of phage on susceptible bacteria may actually result from the detection of some of the phages that have been inoculated but did not adsorb to a host cell yet. In the following, we prefer to focus on the analysis of the presence/absence of phage able to replicate on different types of resistant hosts (i.e. host A, B or AB) because it provides an unambiguous measure of the probability of pathogen adaptation to host immunity.

**Figure 4.**
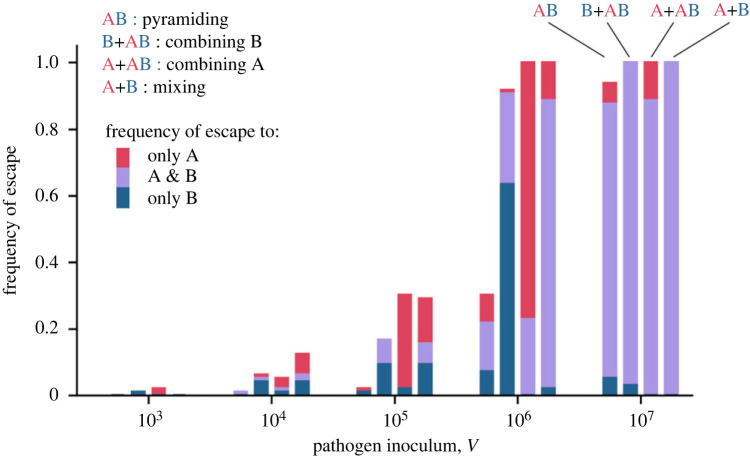
Probability of pathogen evolutionary emergence for different inoculum dose (V) and for different resistance treatments (mixing, combining and pyramiding) in biological assays. We plot the proportion of populations (among the 96 experimental replicates) that resulted in a virus amplification on different resistant hosts. For each pathogen inoculum size *V* we show measures of pathogen evolutionary emergence for the four experimental treatments (from left to right): (i) pyramiding AB, (ii) combining: B + AB, (iii) combining: A + AB, (iv) mixing: A + B. The size of the coloured bars measure to the frequency of emergence of pathogens which could infect only resistant hosts A (red), only resistant hosts B (blue) and both A and B (purple).

**Figure 5.**
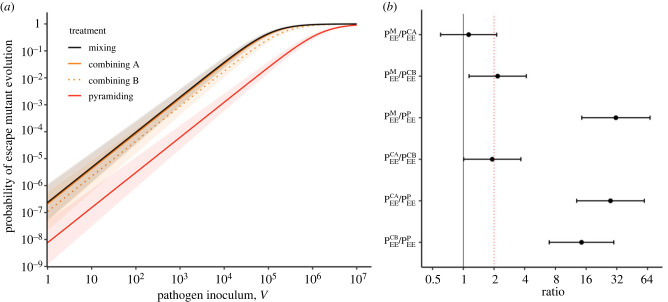
Probability of evolutionary emergence is higher in the *mixing* treatment and lowest in the *pyramiding* treatment in biological assays. (*a*) We plot here the estimation of the probability of evolutionary emergence (i.e. the probability to evolve at least one escape resistance) against the inoculum size *V* and the resistance treatment. The model can be written as $\mathrm{logit}(P_{EE}^{T})\;\tilde{\;}a_{T}\;\log(V)\;+\;b_{T}$, where the slope parameter is the same for all treatments (see electronic supplementary material). The lines indicate the prediction of the statistical model for the different treatments and the shaded areas show 95% confidence interval. (*b*) We compare the estimated values of $b_{T}$ (i.e. the probability of evolutionary emergence of treatment *T* for an inoculum V=1) for all pairs of treatment and we plot . The error bars show 95% confidence interval and the red dashed line refers to a twofold difference in the probability of emergence. This twofold effect is expected when we compare the mixing and the combining treatments ($P_{EE}^{M}/P_{EE}^{CA}$ and $P_{EE}^{M}/P_{EE}^{CB},$see equation (3.1)).

Our analysis of the probability of the phage to adapt to at least one type of resistance confirms our predictions on the effect of inoculum and host composition (figures 4 and 5). In particular, we recover the predicted relationship $P_{\mathrm{EE}}^{M}>P_{\mathrm{EE}}^{C}>P_{\mathrm{EE}}^{P}$ when we focused on the *Combining B* treatment (i.e. a combination of strains B and AB): Tukey Honestly Significant Difference (HSD) test, $P_{\mathrm{EE}}^{M}-P_{\mathrm{EE}}^{\mathrm{CB}}=0.78$, *z* = 3.08, *p* = 0.011; $P_{\mathrm{EE}}^{\mathrm{CB}}-P_{\mathrm{EE}}^{P}=2.67$, *z* = 9.40, *p* < 0.001. However, we find no significant differences between the probabilities of viral evolution in the *Mixing* and in the *Combining A* treatments (i.e. a combination of strains A and AB): Tukey HSD test, $P_{\mathrm{EE}}^{M}-P_{\mathrm{EE}}^{\mathrm{CA}}=0.12$, *z* = 0.49, *p* = 0.96. This suggests that the probability for a virus of acquiring an escape mutation against resistance A is higher than against resistance B (see also Table S3). Note that the expected twofold increase in the probability of viral evolution in the *Combining* treatment relative to the *Mixing* treatment (see equation (3.1)) lies in the 95% confidence intervals we compute: $P_{\mathrm{EE}}^{M}/P_{\mathrm{EE}}^{\mathrm{CA}}=1.13[0.60;2.13]$; $P_{\mathrm{EE}}^{M}/P_{\mathrm{EE}}^{\mathrm{CA}}=2.17[1.14;4.15]$ (figure 5*b*, red dashed line).

Interestingly, similar treatment effects were found when we analysed the ability of phage 2972 to acquire both escape mutations (electronic supplementary material, figure S4). In particular, we found that the *Mixing* treatment was most favourable for the emergence of double escape mutations (Tukey HSD test, $P_{{\mathrm{EE}}_{2}}^{M}-P_{{\mathrm{EE}}_{2}}^{\mathrm{CA}}=1.75$, *z* = 6.71, *p* < 0.001; $P_{{\mathrm{EE}}_{2}}^{M}-P_{{\mathrm{EE}}_{2}}^{\mathrm{CB}}=0.84$, *z* = 3.39, *p* = 0.0039; $P_{EE_{2}}^{M}-P_{EE_{2}}^{P}=1.78$, *z* = 6.81, *p* < 0.001), even if none of the bacteria carry both types of resistance in this treatment. This effect likely results from the sequential acquisition of multiple mutations, which is facilitated in the mixing treatment. In other words, the *mixing* strategy is far less durable than the *pyramiding* strategy. Besides, as predicted by our theoretical model, the probability of evolutionary emergence under the *combining* strategy falls in between the two other strategies and confirms that the presence of single-resistant genotypes speeds up the acquisition of escape mutations and promotes evolutionary emergence even when some hosts are multiresistant.

## 4. Discussion

In this study, we have explored the influence of several factors such as pathogen life history traits (birth and death rates), mutation rates, pathogen initial inoculum size, fraction and depth of host resistance, on the ultimate fate of a pathogen introduced in a heterogeneous host population. In particular, we showed that larger inoculum size favours the emergence and the adaptation of the pathogen to the host population because of two main effects. First, larger inoculum size increases the probability of the introduction of a pre-existing escape mutation which further increases the evolutionary potential of the pathogen population. Second, even in the absence of pre-existing mutations in the inoculum, a larger inoculum size of the wild-type pathogen provides more opportunities for the emergence of escape genotypes by mutation.

Our theoretical analysis yielded clear predictions on the effect of the host composition on the probability of evolutionary emergence of a pathogen: *pyramiding* is the most effective way to reduce the risk of pathogen adaptation, even in the presence of pre-existing escape mutants in the pathogen inoculum (figure 2). The worst strategy is the fully asymmetric *mixing* strategy (e.g. $f_{A}=1$) because it takes only a single escape mutant to exploit the whole host population. The fully symmetric *mixing* strategy is better than the asymmetric *mixing* strategy because, as shown by previous studies, higher host diversity reduces the probability of evolutionary emergence [20,24,33–35]. The efficacy of the *combining* treatment is intermediate and is very sensitive to the relative proportion of single and multiple resistances. In particular, we showed that the overlap between the resistance genes carried by single- and double-resistant host genotypes in the *combining* treatment may greatly enhance the risk of evolutionary emergence because escaping single resistance may provide a ‘stepping stone’ towards the acquisition of multiple escape mutations. We show in the electronic supplementary material, how these theoretical predictions can be generalized with more than two resistance genotypes (equations S11 and S12). In particular, we confirm that larger resistance diversity decreases the probability of evolutionary emergence in the *mixing* strategy [20], and the durability of the *pyramiding* strategy increases with the accumulation of resistances.

Our experimental results confirmed both the positive effect of larger inoculum size and the hierarchy in the efficacy of different host treatments on the probability of pathogen adaptation. Note, however, that our model oversimplifies several features of the pathogen dynamics taking place in our biological experiments. First, we modelled viral growth as a ‘birth-death’ process while the reproduction of a virulent phage follows a ‘burst-death’ life cycle. The burst-death process is expected to alter the variance associated with the reproduction event and may thus alter the predictions of the evolutionary outcome [36], but see [20] for a comparison between these two ways to model pathogen dynamics. Second, we assumed the fraction of the different host genotypes to be constant throughout the experiment. This is a very rough approximation because the fraction of the susceptible hosts will drop relatively rapidly when the wild-type genotype spreads. Consequently, the fraction $f_{R}$ of resistant hosts is expected to increase rapidly through time. Similarly, the relative fraction of the different types of hosts is expected to vary with time after the emergence of single escape mutations that will exploit specifically a fraction of these resistant hosts. Yet, the good match between our theoretical predictions and our experimental results suggests that the conclusions of our model are robust to the specific details of the epidemiology of the pathogen.

Our conclusions are also consistent with a review of the available empirical studies on the durability of different crop protection programs which concluded that *pyramiding* is the most durable strategy [8]. For instance, the durability of wheat cultivars was associated with the pyramiding of multiple resistant genes [7]. A few experimental studies have tracked the evolution of pathogens over several generations and demonstrated the beneficial impact of the *pyramiding* strategy. A study on the evolution of a plant-parasitic nematode showed that the use of pyramided genotypes protected the plant crop over several years [37]. Another study on transgenic broccoli plants indicated that the expression of multiple *Bacillus thuringiensis* (Bt) toxins hampered the epidemiology and evolution of a major insect pest, the diamondback moth (*Plutella xylostella*) [38–40]. In addition, this latter study revealed the detrimental effect of combining the same resistance genes in different plant varieties for the durability of resistance. Indeed, as in our *combining* strategy, the advantage of using plant genotypes containing two dissimilar Bt toxin genes for resistance management may be compromised if they share similar toxins with single-gene plants that are deployed simultaneously.

## 5. Conclusion and broader implications

Microbes carrying CRISPR-Cas immunity against virulent bacteriophages provide ideal biological models to obtain experimental measures of the probability of pathogen emergence and evaluate their ability to escape host resistance under different control strategies [20]. Besides, the specificity of CRISPR-Cas immunity to bacteriophages is very similar to the classical gene-for-gene model of specificity driving the coevolution between many plants and their pathogens. Our biological experiments confirm our theoretical predictions on the influence of (i) the resistance strategy and (ii) the initial dose of the pathogen. In particular, we find that the *pyramiding* strategy is a more effective way to reduce the evolutionary emergence of the pathogen. These microbiological assays confirm that exposing pathogens to a mix of different host genotypes carrying a low number of resistance genes facilitate the adaptation of the pathogen because it provides multiple routes (with slower slopes) towards complex pathogen genotypes carrying multiple escape mutations. This result does not conflict with the positive effect of host diversity for the reduction of pathogen evolutionary emergence [20,24]. But for a given amount of host resistance diversity, the present study shows that stacking this diversity in a limited number of genotypes is a more effective strategy than using a mixture of single-resistance host genotypes to prevent pathogen emergence. The durability of the pyramiding strategy stems from the low probability of multiple escape mutations or on the rare occurrence of single mutations of large effect that may enable a pathogen to escape multiple resistance in one go [41]. The success of the pyramiding strategy may explain why many microbes carry several genes coding for distinct defence systems in their genome [42,43]. While these genomic defence islands may provide immunity against a wide variety of diverse phages, they may also limit the emergence and evolution of bacteriophage variants, thereby increasing the persistence of microbes in various ecosystems.

While our results are relevant for several areas, including for crop management in agriculture [8,10,37] as well as in food fermentation [44], they may also hold for the management of drugs and vaccines. In human immunodeficiency virus (HIV), for instance, the success of the combination therapy is arguably due to the use of the *pyramiding* strategy where the patient is treated simultaneously with multiple drugs [45–47]. A similar conclusion was reached with a theoretical model that explored alternative treatment strategies against bacteria as a combination therapy (*pyramiding*) outperforms other ways to use available antibiotics [48–50]. In malaria, the use of artemisinin-containing combination therapies (*pyramiding*) is also believed to provide a way to slow the spread of antimalarial drug resistance [51–54]. These results also suggest that the use of phage cocktails in phage therapy is likely to be more effective because the pathogenic bacteria will have difficulties to evolve resistance against multiple phages [55,56].

The durability of vaccines may also be explained by the *pyramiding* effect. Unlike therapeutic drugs, some vaccines often elicit multiple immune responses against several pathogen targets and this could explain why resistance to vaccines evolves usually more slowly than drug resistance [57]. The recognition of the value of immune diversity could lead to new vaccination strategies. For example, the deployment of different vaccines among different individuals to create a mosaic of vaccination has the potential to outperform conventional vaccination [58]. Moreover, several studies demonstrated that combining multiple immune responses to different epitopes can increase significantly the efficacy of vaccination [59–63]. The rise of mRNA vaccines [64,65] may facilitate the development of such new generation of multivalent vaccines that could use the pyramiding effect to increase their durability.

Pyramiding multiple defences in the same host may thus provide a durable strategy for both prophylactic and therapeutic control of infectious diseases. The recognition of the value of *pyramiding* is ancient [66] but we hope our work clarifies the complex interplay between demography, evolution and stochasticity. The influence of many other factors remains to be investigated. For instance, our model does not account for the change in the composition of the host population after the start of a viral epidemic. Time-inhomogeneous branching process models could be developed to better understand the influence of these epidemiological feedbacks on evolutionary emergence. In addition, the importance of epistasis in fitness among the escape mutations carried by the pathogen is expected to affect the probability of emergence (see electronic supplementary material, figure S1). Patterns of epistasis are also likely to have an impact on the influence of pathogen recombination [67,68]. Yet, our model does not allow for coinfections and, consequently, does not allow for recombination. The influence of genetic recombination on the robustness of the *pyramiding* effect remains to be investigated. Finally, our joint theoretical and experimental study could be extended to explore a wider range of deployment strategies in space and time [47,69–72]. This approach could thus be used to identify and to test the durability of new strategies to limit the emergence and the evolution of pathogens.

## Data Availability

Data has been deposited in zenodo [73]: https://zenodo.org/records/8114168. Supplementary material is available online [74].
